# Spanning External Fixation for the Treatment of Open Joint Injuries in Pediatric Burn Patients

**DOI:** 10.7759/cureus.2484

**Published:** 2018-04-16

**Authors:** Kelly Carmichael, Daniel Torres, Carlos J Jimenez, Matthew C Comley, Ernest B Evans

**Affiliations:** 1 Department of Orthopaedic Surgery and Rehabilitation, University of Texas Medical Branch; 2 LVPG Orthopedics and Sports Medicine–bethlehem; 3 Department of Surgery, University of Texas Medical Branch; 4 Department of Orthopaedic Surgery and Rehabilitation, The University of Texas Medical Branch

**Keywords:** orthopedics, external fixator, children, open joint burns

## Abstract

In this article, we report a case series on spanning external fixation for the treatment of open joint burn injuries in a pediatric population. We reviewed the case logs of all orthopedic surgeons from 2000 to 2010 at a burn hospital to identify pediatric patients with open joints secondary to burn injuries.

Nine patients who sustained open joint injuries after a burn and treated with a spanning external fixator (SEF) were identified. Characteristics of the burns included: five elbow, four knee; seven flame, two electrical; average total body surface area affected 49.4% (range 25%–79%); substantial third-degree burn in all patients. Average age at the time of the burn was 8.6 years (range: 2 months–17.9 years). Average time from the burn to SEF placement was 7.1 weeks (range: 3–10.5 weeks). Before SEF placement, an average of 3.8 skin grafting procedures (range: 1–7) were performed to treat the open joint injuries. SEFs remained in place for an average of 6.4 weeks (range: 3–9 weeks). After SEF application, skin graft procedures were performed on average 0.6 times (range: 0–3). There were two complications (22%) considered to be directly associated with the SEF procedure due to the failure of fixation.

Placement of an SEF for an open joint burn injury in children is an effective means to treat these uncommon and difficult injuries. We recommend early SEF to help assist with soft tissue healing and decrease the number of skin grafting procedures in this population.

## Introduction

Skeletal immobilization is one of the oldest and most used treatment modalities for the trauma patient. Burn care has evolved from early skeletal traction with full body elevation and delayed surgical care [1]. Now, early excision of the burn injury with skin graft application and early immobilization is employed. Early excision with skin graft application decreases the amount of soft tissue-induced contracture deformity with eventual improved functional recovery [2,3].

Today, early excision of third-degree burns with skin graft application provides fast wound closure with good functional outcomes [2]. Nosocomial problems like prolonged mechanical ventilation, prolonged hospital stay and soft tissue infections are decreased with expedited surgical management and early immobilization.

Early surgical removal of burn tissue with skin graft application is used to decrease the amount of contraction and scar formation, providing better functional and cosmetic results [3,4]. Skin grafting alone, however, is not enough to treat the complicated/unstable open joint with overlying third-degree burn injury. The use of limited (segmental) external fixation has proven to be effective in providing bone stability and a stable bed for skin graft application. Early fixation reduces pain and discomfort and improves graft take, potentially reducing the amount of surgical interventions needed. Advances in burn resuscitation, tissue banking techniques and blood transfusion protocols allow for safe early surgical management of burn patients.

When orthopedic intervention is needed in the pediatric burn population, it is usually because of burn sequelae, such as burn contractures or heterotopic ossification [5-8]. We utilize a multispecialty approach whereas burn surgeons, plastic surgeons, and orthopedic surgeons are involved with complex burn injuries [2-4,9]. We have identified an acute pediatric burn injury that may require early orthopedic intervention, namely, open joints after a burn injury that involves the elbow or the knee. The orthopedic treatment comes in the form of a spanning external fixator (SEF). To our knowledge, there have been no reports describing SEF for treatment of open, major joint burn injuries in a pediatric population. In our experience, skin grafts take poorly in open joints. We hypothesized that the use of SEF will improve skin graft incorporation and is necessary for the treatment of open joints in burn patients.

## Case presentation

We performed a retrospective chart review at our pediatric burn hospital to examine the results of open, major joint burn injuries after SEF. The study was approved by the Institutional Review Board. Our inclusion criterion was any SEF that was performed for an open, major joint burn injury not in the hand or foot. Our exclusion criteria were any fractures or dislocations without the use of the SEF or use of an SEF in chronic injury/sequelae or placement in the hand or foot.

**Figure 1:**
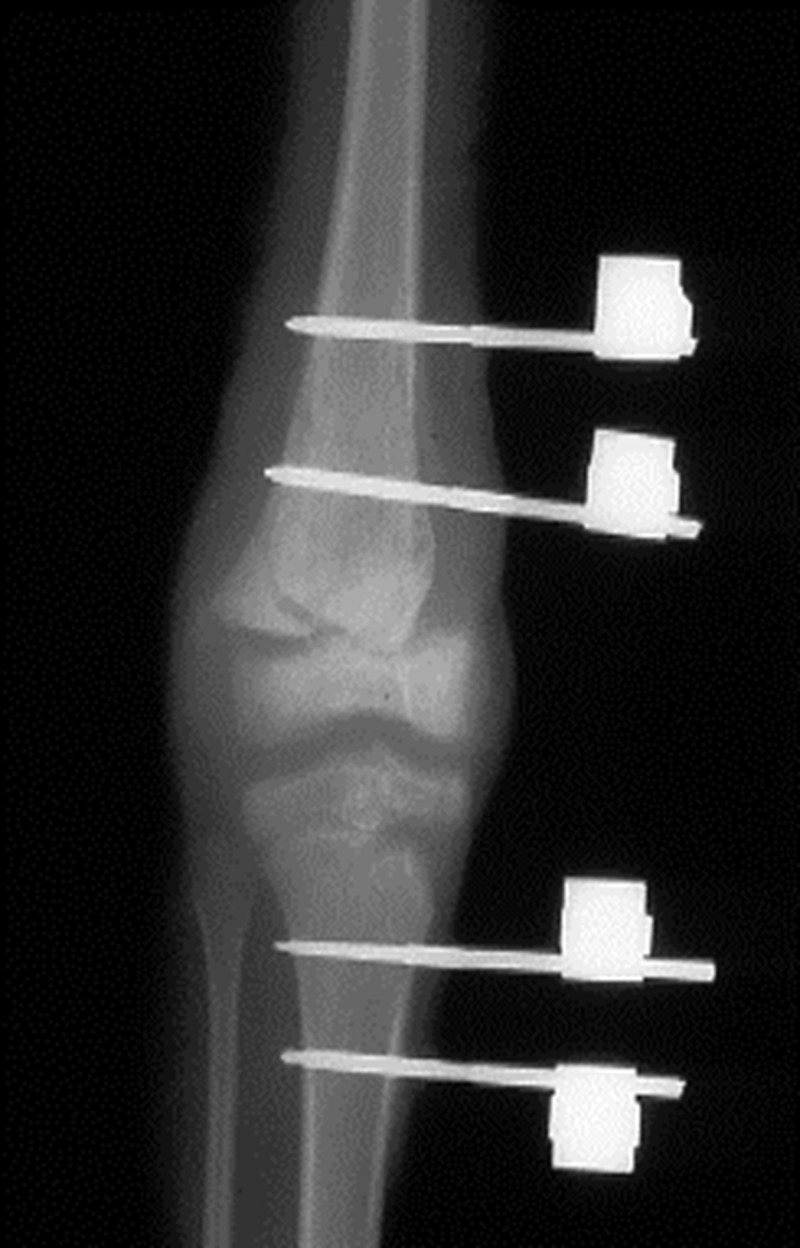
Radiograph showing spanning external fixator in place.

We performed a search of all procedures performed from 2000 to 2010 by using all external fixation CPT codes (20690, 20692, and 20694). Because of the low numbers of eligible charts encountered with CPT codes, we decided to do a case log review of all orthopedic surgeons from 2000 to 2010. Once inclusion and exclusion criteria were met, the patients’ charts were reviewed in detail. We collected demographic data that included age at the time of injury, sex, mechanism of injury, and degree of burn. Other data collected included the affected joint, the type of hardware used for the SEF, length of time from injury to placement of SEF, amount of time that the SEF was left in place, the amount and type of skin grafting procedures that were performed before and after SEF, and complications. Figure 1 shows an example of a radiograph with SEF in place.

We also looked at the rehabilitation of the affected joint. Physical therapy (PT)/occupational therapy (OT) notes from inpatient and outpatient encounters were reviewed in detail. Range of motion (ROM) and strength were recorded.

A total of 182 burn patients were identified to have had orthopedic interventions. Of these, only 76 patients had external fixators placed. Only nine of these patients had fixators used to treat open, major joint injuries after a burn as shown in Table 1. Five presented with open elbow injury, and the other four presented with open knee injury. Of the latter, two also presented with joint instability. The fixators were placed without joint distraction. Both elbow and knee were placed in full extension by convention. We did not study fixation in any other degrees of flexion other than full extension. Average age at the time of the burn was eight years, seven months (range: 2 months–17 years, 9 months). Average total body surface area (TBSA) affected was 49.4% (range: 25%–79%). Seven patients had flame injury, and two had electrical injury. One of the flame injuries also had electrical injuries. All injuries were mostly third-degree burn.

**Table 1:** Patient demographics, injury, and SEF treatment. SEF: Spanning external fixator; TBSA: Total body surface area. * Patient had unaccounted procedures done in Mexico before transfer to our hospital.

Patient	Age at the time of injury	Sex	TBSA	Degree of burn	Injury mechanism	Time before SEF placement	Skin grafting surgeries before SEF	Time in Ex-Fix	Skin grafting surgeries after SEF
1	13 months	F	73%	3°	Flame	6.5 weeks	4	3 weeks	1
2	10 years 8 months	M	63%	3°	Flame	8 weeks	7	6 weeks	0
3	4 years 4 months	M	41.5%	3°	Flame	6 weeks	2	4 weeks	0
4	17 years 9 months	M	30%	3°	Flame and electrical	3 weeks	2*	7.5 weeks	3
5	7 years 4 months	M	32%	3°	Electrical	3 weeks	3	8.5 weeks	2
6	11 years 6 months	M	25%	3°	Flame	10.5 weeks	3	9 weeks	0
7	8 years 5 months	M	79%	3°	Flame	8.5 weeks	6	5 weeks	0
8	16 years 6 months	M	48%	3°	Electrical	7.5 weeks	6	6 weeks	0
9	2 months	F	53%	49.5% 3°, 3% 2°	Flame	10.5 weeks	1	8.5 weeks	1

Average time from the burn to SEF placement was 7.1 weeks (range: 3–10.5 weeks). Before SEF placement, an average of 3.8 skin grafting procedures (range: 1–7) was performed to treat the open joint injuries. The spanning external fixators remained in place for an average of 6.4 weeks (range: 3–9 weeks). Skin grafts were placed over the open joint areas at the time of fixator application. An average of 0.6 skin grafting procedures was performed after the external fixation was applied (range: 0–3).

Two complications (22%) were considered to be directly associated with the external fixation procedure. Both were related to loss of fixation requiring revision fixation. Other complications recorded were considered likely due to the nature of the injury (e.g., heterotopic ossification, contractures). Average postoperative follow-up was eight months (range: 2–13 months). Postoperative rehabilitation records were only available for six patients, as shown in Table 2.

**Table 2:** Outcomes after SEF. HO: Heterotopic ossification; LLD: Limb length discrepancy; MCL: Medial collateral ligament; PT: Physical therapy; ROM: Range of motion; SEF: Spanning external fixator; WNL: Within normal limits.

Patient	Complications	Joint involved	Final strength	Final ROM
1	None	Right knee	5/5	WNL
2	None	Right elbow	5/5	Ext/Flex: 30–110° Sup/Pron: 30°–WNL
3	Elbow contracture	Right elbow	5/5	Ext-Flex 0–119° Sup/Pron: 69–80°
4	SEF revision; continued weakness	Right elbow	Flex: 4/5 Ext: 2/5 Pro-Sup: 1/5	Ext-Flex 0–110° Sup/Pron: 30–70°
5	Bony prominence that needed resection	Left knee	No data	No data
6	Severe damage to the MCL and spontaneous fusion, LLD and pain	Left knee	No data	No data
7	Residual stiffness and weakness	Right knee	3+/5	Ext-Flex 0–45°
8	Ulnar nerve neuropathy, elbow stiffness and HO	Right elbow	4-/5	Ext-Flex 20–90° Sup/Pron: 25–85°
9	Elbow contracture, SEF revision	Left elbow	No data	No data

Patient 1 was a 13-month-old female who sustained a flame injury from a house fire. She had 73% TBSA third-degree injuries. Her right knee had an open joint injury that was treated with four skin grafting procedures prior to SEF placement. SEF was placed 46 days after the injury and was kept on for three weeks. K-wires and a 140-mm bar were used as a micro-SEF system. The patient required one additional skin grafting procedure to her right knee after SEF was in place. She had an eight-month follow-up after the injury and her PT/OT notes reported ROM within normal limits (WNL) and a 5/5 motor strength. She did have continued joint instability after SEF that required eight weeks of knee immobilizer use. However, no long-term complications requiring additional surgeries were recorded.

Patient 2 was a 10-year-8-month-old male who sustained a flame injury after a motor vehicle accident (MVA). He had 62.5% TBSA third-degree injuries. His right elbow had an open joint injury that was treated with seven skin grafting procedures prior to SEF placement. SEF was placed 55 days after the injury and was kept on for six weeks. Shantz pins and bars were placed as the external fixator system; this was locked in full extension. The patient required no additional skin grafting procedures to his right elbow after SEF placement. He had a 10-month follow-up after the injury and his PT/OT notes documented a final ROM of 30–110° in the extension and flexion arc, supination of 30° and pronation WNL, and a 5/5 motor strength. No other complications were recorded for this patient.

Patient 3 was a 4-year-4-month-old male who sustained a flame injury from a house fire. He had 41.5% TBSA third-degree injuries. His right elbow had an open joint injury that was treated with two skin grafting procedures prior to SEF placement. SEF was placed 43 days after the injury and was kept on for four weeks. Shantz pins and bars were placed using the Synthes medium external fixator system; this was locked in full extension. The patient required no additional skin grafting procedures to his right elbow after SEF placement. He developed an elbow contracture that was treated with an antecubital soft tissue plastic surgery contracture release. Prior to his surgical release, he had an ROM of 0–25° in the extension and flexion arc, supination of 0°, and pronation 0°. He had a 13-month follow-up after the injury and his PT/OT notes reported a final ROM of 0°–119° in the extension and flexion arc, supination of 69° and pronation 80°, and a 5/5 motor strength. His only complication was the elbow contracture.

Patient 4 was a 17-year-9-month-old male who sustained a flame and electrical injury after an MVA involving a power line. He had 30% TBSA third-degree injuries. His right elbow had an open joint injury that was treated with two skin grafting procedures prior to SEF placement. Other unknown procedures were performed in Mexico before transfer to our institution. SEF was placed 23 days after the injury and was kept on for 7.5 weeks. Four-millimeter Shantz pins and bars from the Synthes small external fixator set were placed; the fixator was secured in full extension. The patient had an SEF failure likely due to under sizing. The fixator was revised with a Synthes large external fixator system eight days after the initial one. The patient required three additional skin grafting procedures to his right elbow after SEF placement. He had a 12-month follow-up after the injury and his PT/OT notes reported a final ROM of 0°–110° in the extension and flexion arc, supination of 30°, and pronation 70°. He did have some continued weakness with elbow flexion at 4/5, extension 2/5, and pronation/supination 1/5 motor strength. His complications were SEF failure and weakness.

Patient 5 was a 7-year-4-month-old-male who sustained an electrical burn involving a power line. He had 32% TBSA third-degree injuries. His left knee had an open joint injury that was treated with three skin grafting procedures prior to SEF placement. SEF was placed 24 days after the injury and was kept on for 8.5 weeks. Shantz pins and bars were placed as the external fixator; this was locked in full extension. The patient required two additional skin grafting procedures to his left knee after SEF placement. He also required a bony prominence resection while in SEF. He had no PT/OT notes or follow-up recorded. He had no significant complications.

Patient 6 was an 11-year-6-month-old male who sustained a flame burn from an MVA. He had 25% TBSA third-degree injuries. His left knee had an open joint injury that was treated with three skin grafting procedures prior to SEF placement. SEF was placed 74 days after injury and was kept on for nine weeks. Shantz pins and bars were placed as the external fixator system; this was locked in full extension. He required no additional skin grafting procedures to his left knee after SEF placement. The patient had no ROM measurements recorded because the left knee was immobilized in full extension for a prolonged period of time. Complications were extensive. The patient ended up with significant damage to the medial collateral ligament and the medial femoral epiphysis from the injury developed a spontaneous knee fusion. Because of the growth plate disturbances, he developed a varus deformity and leg length discrepancy of 4.0 cm which caused persistent pain. He ended up requiring an osteotomy and Ilizarov placement for alignment correction and lengthening.

Patient 7 was an 8-year-5-month-old male who sustained a flame burn from an MVA. He had 79% TBSA third-degree injuries. His right knee had an open joint injury that was treated with six skin grafting procedures prior to SEF placement. SEF was placed 61 days after the injury and was kept on for five weeks. An SEF was placed using the Smith Nephew Richards system with 5.0-mm Shantz pins and bars. The patient required no additional skin grafting procedures to his left knee after SEF placement. He had a 10-month follow-up after the injury and his PT/OT notes recorded a final ROM of 0°–45° in the extension and flexion arc. His last recorded motor strength was 3+/5. His complication was residual knee stiffness and weakness.

Patient 8 was a 16-year-6-month-old male who sustained an electrical burn from contact with a power line. He had 48% TBSA third-degree injuries. His right elbow had an open joint injury that was treated with six skin grafting procedures prior to SEF placement. SEF was placed 53 days after the injury and was kept on for six weeks. An SEF was placed using the Smith Nephew Richards system with 5.0-mm Shantz pins and rods. The patient required no additional skin grafting procedures to his left knee after SEF placement. However, he did require two additional surgeries after the SEF was removed because of complications, including one surgery that incorporated an ulnar nerve transposition, heterotopic ossification removal, and contracture release with arthrofibrosis excision. The second surgery was a flap to cover soft tissue deficit that occurred after the elbow release. The patient had a 13-month follow-up after the injury and his PT/OT notes reported a final ROM of 20°–90° in the extension and flexion arc, supination and pronation were not tested, and a 4-/5 motor strength. Prior to his elbow contracture release, he had an ROM of 0°–30° in the extension and flexion arc, supination of 25° and pronation 85°, and motor strength was not tested. Complications included heterotopic ossification, cubital tunnel syndrome, and elbow stiffness.

Patient 9 was a 2-month-old female who sustained a flame burn from a house fire. She had 49.5% TBSA third-degree injuries and 3% TBSA second-degree injuries, 53% total. Her left elbow had an open joint injury that was treated with one skin grafting procedure prior to SEF placement. SEF was placed 74 days after the injury and was kept on for 8.5 weeks. Three-millimeter Shantz pins and rods were placed as an external fixator. The patient required no additional skin grafting procedures to her left elbow after SEF placement. However, she did require an SEF revision because the initial fixator was pulled out. She was lost to follow-up owing to a major natural disaster and transfer to a different hospital. The patient did develop some elbow stiffness and contracture at the time of SEF removal; however, her final ROM and strength are unknown.

## Discussion

Orthopedic intervention in the acute setting for pediatric patients with burn injuries is not common. Skin graft healing around a moving joint may be difficult to achieve, as too much soft tissue shearing force occurs as the joint is ranged. In our experience, immobilization using splints of bivalve casts resulted in skin grafts not incorporating well around open joints. Skeletal traction, fixation or both in the acute setting of the burn population are not a novel idea [1]. Historically, the approach was used to help prevent joint contractures. However, this required prolonged periods of hospitalization and immobilization, which in turn created their own set of complications.

For most acute burn injuries around hinged joints, such as the knee and elbow, soft tissue deficits can be treated with simple skin grafting. Open joints and larger deficits and/or more complex deficits may require plastic surgery flap type reconstructions [3,4,9]. Skin grafts and soft tissue procedures for the elbow and knee can be challenging, as they sometimes do not incorporate when there is excessive motion. Immobilization with simple splints may not be an option, as the surgical sites may need daily monitoring and wound care. We believe splints also allow enough motion to disrupt graft incorporation.

Although open joints are rare, we were able to present a small case series on the placement of an SEF for an open joint to offer this as a technique and treatment option. This series demonstrated that external fixation is a viable option to treat these injuries.

The amount of autograft available is limited, especially in pediatric patients (with an average of 49.4% TBSA burn in our series). Therefore, it is imperative to avoid skin graft failure. We feel this technique can help limit the number of skin grafts required to achieve treatment of the open joint and bone. The one skeletally mature patient (patient 4 at almost 18 years old) was removed, then only one skeletally immature patient in the series required any additional grafting after the SEF; so the number of skin grafts required for these pediatric patients dropped to 0.2 (0–2). The complication rate directly associated with the external fixator was 22%. Given the serious nature of these injuries, we feel the outcomes were satisfactory after SEF. We acknowledge the number of patients in this study is inadequate to draw any definitive conclusions. We do feel that SEF shows promise in improving the care of burn patients with open joints.

## Conclusions

In pediatric patients with open elbow or knee joints after a burn injury, we recommend early SEF to help assist with soft tissue healing and feel it may decrease the number of skin grafting procedures. We seek and encourage future efforts to design prospective study protocols with early use of segmental external fixation devices and evaluating graft take ratios, pain, and functional outcomes. Positions other than full extension of the joint need to be studied to see if this will improve range of motion and function of the joint.
